# When the image loses its shape: a comparative study of college students' understanding pathways of abstract and figurative art

**DOI:** 10.3389/fpsyg.2026.1762388

**Published:** 2026-05-18

**Authors:** Shaowen Wang, Wenbin Li, Le Yin, Mengling Lyu

**Affiliations:** Sichuan University Jinjiang College, Meishan, China

**Keywords:** abstract art, contextual factors, figurative art, mixed-methods, pathways of understanding

## Abstract

This study examines path differences in abstract and figurative art comprehension between art and non-art majors among college students. Employing a phased mixed-methods approach, the qualitative phase utilized semi-structured interviews and thematic analysis to develop a “perception-emotion-context-reasoning/narrative-interpretation” comprehension model, which informed the creation of a quantitative scale. The quantitative phase employed this model as a framework for reliability and validity testing, alongside multi-group path analysis. It compared and refined the model across four scenarios (“abstract/concrete × art/non-art”). Results indicate that contextual factors play a pivotal “hub-and-spoke” role in artistic comprehension. Artistic groups leverage context to engage in abstract reasoning and narrative construction, producing symbolic and academic expressions. Non-artistic groups, however, are often emotion-driven or rely on subjective speculation, leading to everyday associations or comprehension barriers. Understanding abstract works relies on perception and emotion, which, through context, ascend to the symbolic level. Concrete works, supported by context, activate narrative and linguistic pathways. The study provides mutual validation across qualitative and quantitative dimensions, proposing pedagogical and exhibition insights such as tiered contextual cues, narrative scaffolding, and task-oriented observation. These approaches facilitate the transition from experiential to professional understanding, enriching cognitive processing theories of art comprehension and offering an actionable framework for aesthetic education and museum practice.

## 1 Introduction

Within the contemporary art discourse, abstract and figurative art—as two primary expressive paradigms—have long sparked extensive debate. Figurative art, grounded in representational imagery, offers viewers an intuitive path to understanding through explicit narrative threads and visual representations, rendering it more accessible to the general public. Abstract art, conversely, transcends representational boundaries, emphasizing the autonomy of form, color, and structure, with its meaning generation dependent upon viewers' personal experiences and intuitive interpretations. This distinction often leads to abstract art being perceived as “obscure and difficult to understand,” while figurative art tends to evoke stronger emotional resonance. College students constitute a vital segment of future cultural audiences and represent a critical group in the development of aesthetic cognition. Therefore, comparing their differing pathways to understanding these two art forms holds both academic value and provides significant reference for art education and exhibition practices.

Art appreciation is generally understood as a complex process interweaving cognition, emotion, and cultural experience. Meaning is not inherent in the artwork itself but dynamically generated through viewer engagement (Jauss, 1982; Gadamer, 1989). In this process, figurative art more readily activates viewers' existing schemas and semantic associations, thereby facilitating narrative interpretation and semantic processing (Paivio, 1986; Hemati and Hossein-Zadeh, 2018). In contrast, abstract art relies on higher-order intuitive processing and emotional engagement, featuring open and diverse meaning construction that manifests as more individualized neural processing patterns (Durkin et al., 2025; Chmiel and Schubert, 2019). Further research indicates that disciplinary background and artistic training significantly influence interpretive approaches. Systematic art education reduces cognitive load while enhancing formal analysis and academic interpretation; conversely, untrained viewers tend to rely more on emotional responses and life experiences for meaning-making (Bîzoi and Bîzoi, 2025; Gurung and Herrboldt, 2024; Schepman and Rodway, 2021a). Consequently, art majors typically begin with form, composition, and symbolic systems before progressing to abstract or academic narratives, whereas non-art majors lean toward emotional associations grounded in feelings and personal experiences (Schepman and Rodway, 2021b). Overall, artistic understanding constitutes a dynamic process of construction from perception to interpretation. However, existing research predominantly focuses on comparative outcomes rather than revealing the internal pathways and mechanisms of “how understanding is generated.” Specifically, the following shortcomings persist: First, studies predominantly concentrate on artists or the general public, with relatively insufficient research on university students and their internal variations. Second, existing studies predominantly examine outcome variables such as preferences, acceptance levels, or emotional responses, neglecting the phased and mechanistic nature of the comprehension process. Third, methodologies remain dominated by single quantitative or qualitative approaches, lacking cross-group comparisons and dynamic tracking designs. Consequently, we currently lack a thorough understanding of how different types of artworks are progressively perceived, interpreted, and internalized by university student audiences. This study aims to address these gaps by exploring the pathways of understanding and their variations when university students appreciate abstract and figurative art.

Focusing on university students, this research compares the differences in abstract and figurative art comprehension pathways between art majors and non-art majors. Employing a “qualitative exploration—quantitative validation” design, it first uses interviews and thematic analysis to identify students' perceptual, emotional, and interpretive stages during viewing, thereby exploring primary comprehension pathways. Subsequently, an analytical framework is constructed based on qualitative findings, with group differences validated through questionnaires and statistical methods. This study seeks to reveal universal patterns in the comprehension process and the role of disciplinary background, shifting art psychology and aesthetics research from outcome-oriented to process-oriented approaches. It provides empirical references for art education and exhibition planning, fostering deeper audience understanding and acceptance of abstract art.

## 2 Theoretical background and literature review

### 2.1 Receptor theory perspective

Receptor theory, proposed by Jauss (1982) and Gadamer (1989), emphasizes that artistic meaning emerges through audience participation. Neuroaesthetic research indicates abstract art stimulates stronger individualized brain activity, suggesting audiences incorporate more personal associations during interpretation (Durkin et al., 2025). Cross-cultural studies further validate the dynamic nature of meaning generation. Fan (2025) noted that British audiences' understanding of Chinese theater is influenced by cultural memory. Wibawa et al. (2023) discovered Balinese patterns acquiring new meanings in contemporary contexts. Yu (2024) revealed dialect differences leading to varied interpretations of the novel Splendid Flowers. This theory has now extended to digital art, with Tao (2024) arguing that meaning construction in AI literature relies on readers' engagement between “disenchantment” and “re-enchantment.” Hidayat et al. (2024) found that virtual influencers lacking human characteristics reduce audience acceptance. Overall, artistic meaning is actively constructed by audiences within their experiential and cultural frameworks, with abstract art particularly requiring audience subjectivity (Durkin et al., 2025; Tao, 2024).

### 2.2 Cognitive psychology perspective: comparative analysis of perceptual mechanisms

Cognitive psychology reveals differences in the mechanisms underlying the perception of abstract vs. figurative art. Gestalt psychology posits that the human brain tends to integrate fragmented stimuli into a unified whole (Wertheimer, 1923). When viewing ambiguous images, viewers automatically complete the imagery (Silva-Gago et al., 2025), indicating that pattern recognition is the core process of artistic perception, influenced by both culture and technology (Üstün, 2024). Dual-Coding Theory (Paivio, 1986) posits that cognitive processing involves linguistic and visual channels. Figurative art activates dual-channel coordination, facilitating semantic understanding. Abstract art, lacking nameable objects, relies more on intuitive experience for comprehension (Hemati and Hossein-Zadeh, 2018). Research confirms dual-channel advantages: painting training reduces cognitive load (Bîzoi and Bîzoi, 2025), while text-image integration and immersive narratives enhance memory and deep processing (Gurung and Herrboldt, 2024; Barrett et al., 2025). Cognitive load theory (Sweller, 1988) indicates that comprehension is hindered when information complexity exceeds working memory capacity. Concrete art imposes lower cognitive load due to explicit schemas, whereas abstract art demands greater cognitive resources and higher load. External cues or technological aids (e.g., captions, AR) effectively alleviate this load (Chmiel and Schubert, 2019; Chen and Mokmin, 2024; Tiwari and Bhagat, 2024).

### 2.3 Meaning generation from a semiotic perspective

The semiotic perspective emphasizes the referential differences between abstract and figurative art: figurative art is characterized by clear representation of reality, while abstract art de-emphasizes representation, tending toward symbolism and open expression. Eco (1989) termed this the “open work,” where meaning depends on the audience's participation and construction. Parung et al. (2024) found that students can generate meaning across symbolic systems when translating music into paintings. Empirical research indicates figurative art possesses greater shared meaning, while abstract art elicits more divergent interpretations. Audiences often employ more vivid language when describing abstract works, reflecting active meaning-making (Compagno, 2024). Furthermore, Ding (2024) contends that translating visual elements into language constitutes a complex process of symbolic translation.

### 2.4 Research gaps

In addition to disciplinary background, artistic expertise is widely recognized as a key factor influencing art perception and interpretation. Expertise is not only reflected in formal training but also in visual literacy, familiarity with artistic conventions, and interpretive strategies. Prior research suggests that individuals with higher levels of artistic expertise are more likely to engage in analytical, symbolic, and theory-driven interpretations, whereas novices tend to rely more on emotional responses and personal experiences. In the present study, disciplinary background (art vs. non-art majors) is used as a proxy for differing levels of artistic expertise.

Research indicates that audiences generally prefer representational art, and artistic training enhances comprehension of abstract art (Darda and Cross, 2022). Abstract art activates brain regions associated with imagination, while representational art primarily engages visual recognition (Durkin et al., 2020). Audiences automatically complete ambiguous images (Silva-Gago et al., 2025) and describe abstract works using more concrete language (Schepman and Rodway, 2021b). Cross-media creation and AR technology facilitate multichannel processing, reducing cognitive load in abstract learning (Parung et al., 2024; Chen and Mokmin, 2024; Tiwari and Bhagat, 2024; van Nooijen et al., 2024). Existing research predominantly focuses on outcome differences, lacking dynamic insights into audience comprehension pathways. While questionnaires measure degree of understanding, they struggle to capture meaning-making processes. Consequently, this study employs a mixed-methods approach combining qualitative and quantitative methods to compare university students' understanding pathways in abstract vs. figurative art, thereby addressing research gaps and providing empirical support.

## 3 Research methods

All procedures in this study comply with the Declaration of Helsinki (1964) and its subsequent amendments or comparable ethical standards. The research procedures and instruments were reviewed and approved by the ethics review committee of the authors' affiliated institution on August 29, 2025. Before the commencement of the study, all participants were informed of the nature and purpose of the research and signed informed consent forms, confirming that they voluntarily participated with full understanding of the relevant information. The study was conducted from September to October 2025. All participants took part after being assured of anonymity and being clearly informed that their responses would be used solely for academic purposes. As all participants were adults, parental consent was not required.

### 3.1 Research participants

The qualitative phase employed purposive and maximum diversity sampling, the qualitative phase employed purposive and maximum diversity sampling, recruiting 24 university students (12 art majors and 12 non-art majors). Participants ranged in age from 20 to 22 years, with a gender distribution of 12 males and 12 females. Disciplinary background was used as an indicator of differing levels of artistic expertise, as art majors typically receive systematic training in visual analysis and art theory, whereas non-art majors generally lack such formal training with balanced gender, grade level, and artistic experience. The quantitative phase distributed questionnaires via the Wenjuanxing platform, yielding 732 responses. After excluding invalid and duplicate samples, 665 valid data points were retained, comprising 344 arts students and 321 non-arts students. The participants were sophomore and junior students aged between 20 and 22, including 312 males and 353 females.

### 3.2 Research materials and tools

#### 3.2.1 Research stimuli materials

The experimental materials comprised classic abstract and figurative artworks, specifically representative works by Kandinsky, Rothko, Van Gogh, and Rembrandt. Selection criteria included: representativeness in art history and public perception; comparable visual complexity, color saturation, and compositional scale to avoid confusion from stimulus differences.

#### 3.2.2 Qualitative interview questionnaire

The interview employed open-ended questions structured around the “perception-emotion-context-interpretation” framework. In this study, perception was defined as early-stage visual processing and attentional responses to formal elements of artworks, including color, shape/line, and composition. Accordingly, interview questions were designed to elicit participants' perceptual responses rather than to directly measure perception as a psychological construct. This design aimed to compare the interpretive pathways and disciplinary influences between art and non-art students when engaging with abstract and figurative art. The theoretical framework synthesizes perspectives from reception aesthetics, semiotics, and cognitive psychology (Jauss, 1982; Gadamer, 1989; Eco, 1989; Paivio, 1986; Sweller, 1988; see Supplementary Appendix A for outline).

#### 3.2.3 Quantitative research phase questionnaire

The questionnaire was developed based on qualitative interview findings and the theoretical framework, constructing four dimensions: Perception, Emotion, Comprehension, and Context. After expert review and pre-testing revisions, the final scale comprised 90 items. Perception variables included color attention, shape/line perception, and composition/detail observation. Emotional responses encompass positive, negative, and complex emotions. Comprehension variables include art recognition, narrative construction, abstract reasoning, symbol interpretation, comprehension barriers, academic narratives, and life-related associations. Contextual factors comprise disciplinary background and personal experience. The questionnaire employs a five-point Likert scale and incorporates separate sections for abstract and figurative art within the same instrument to enable both intra-group and inter-group comparisons. (See Supplementary Appendix B for the quantitative research phase questionnaire).

### 3.3 Research procedures and data analysis

This study employs a two-stage mixed-methods design following a “qualitative exploration—quantitative validation” logic. The qualitative phase utilizes semi-structured interviews and thematic analysis to identify key nodes (perception, emotion, context, interpretation) in university students' understanding of abstract and figurative art, establishing a preliminary model. The quantitative phase developed a questionnaire based on this model and conducted a large-sample online survey, employing confirmatory factor analysis (CFA) and structural equation modeling (SEM) for path analysis and multi-group comparisons.

Twenty-four participants were interviewed, each session lasting 30–45 min, with artworks presented under standardized conditions to balance order effects. Transcribed and anonymized recordings were independently coded by two researchers (Cohen's κ ≥ 0.70), forming the “Perception-Emotion-Context-Interpretation” model. In the questionnaire phase, participants viewed abstract and figurative works while completing 45 assessments; 665 valid responses were retained after screening per (Hair et al., 2012) standards. Analysis included reliability and validity testing, SEM path analysis, and multi-group comparisons to validate disciplinary background differences.

This study strictly adhered to academic ethics standards. All participants voluntarily consented after receiving informed consent. Data underwent anonymization and was used solely for academic research to ensure privacy and information security.

## 4 Qualitative research

### 4.1 Open coding results

This section presents the open coding results from the qualitative phase. Through line-by-line analysis of 24 interview transcripts, a total of 283 preliminary nodes were identified. Using NVivo (Lumivero, Denver, CO, USA) 12 and adhering to grounded theory's open coding principles, two researchers conducted independent coding with good agreement (Cohen's κ = 0.78).

Open coding aims to extract concepts from the data without pre-established frameworks. Findings indicate that university students' responses to artworks primarily unfold across four dimensions: Perception, Understanding, Emotion, and Context. Table 1 illustrates typical coding examples, demonstrating the process of refining raw statements into conceptual categories. (Complete open coding results are presented in Supplementary Appendix C).

**Table 1:** Open coding examples.

Respondent	Raw statement	Preliminary code	Dimension category
A3	“The colors in this painting evoke a sense of oppression for me.”	Color and emotion	Perception/emotion
A6	“Judging by the repetition of lines, the artist might be conveying a sense of order.”	Form reasoning	Understanding
N4	“I can't make any sense of it at all; it just looks like random scribbles.”	Comprehension barrier	Understanding
…	…	…	…

### 4.2 Hierarchical node coding results

Building upon the initial open coding phase, this study further organized and synthesized the findings to establish a hierarchical thematic node system. Through continuous comparison of the corpus and conceptual aggregation, the interviewees' expressions were systematically categorized into four top-level categories, each subdivided into several sub-nodes. The specific structure is presented in Table 2.

**Table 2:** Hierarchical node structure diagram.

Top-level category	Subnode	Description
Perception	Color attention	Sensitivity and appreciation of the color of a work.
	Shape/line	Attention to the lines, shapes, and structure of a work.
	Composition/detail observation	Attention to the overall layout and details of a work.
Understanding	Art identification	Identifying a style, genre, or artist.
	Narrative construction	Organizing the content of a work into a coherent story or scene
	Symbolic interpretation	Constructing deeper meaning based on symbols or metaphors
	Comprehension barrier	Confusion or stagnation in interpreting a work. Academic Narrative: interpreting an work within an academic framework such as art history and theory
	Academic narrative	Interpreting an work within an academic framework such as art history and theory
	Subjective speculation	Speculations and hypotheses made when understanding is insufficient.
	Life association	Associations and interpretations based on everyday experience
Emotion	Positive emotion	Positive feelings such as pleasure, enjoyment, and excitement experienced during viewing
	Negative emotion	Negative experiences such as depression and boredom experienced during viewing
	Complex emotion	The audience's experience of complex emotions such as conflict and tension
Context	Disciplinary background	The moderating influence of education and professional training on work comprehension
	Personal experience	Audience reliance on individual life and experiential contexts to interpret works

Research findings indicate that students generally begin their appreciation of art with sensitive perception of formal elements, yet exhibit group differences in comprehension and emotional engagement. Figurative art tends to facilitate intuitive recognition and emotional associations, while abstract art often triggers cycles of confusion and reinterpretation. Background factors (including disciplinary background as a proxy for expertise) significantly moderate the depth of understanding. Art students are more likely to engage in abstract reasoning and academic discourse, while non-art students tend to remain at the level of emotional associations and subjective speculation.

### 4.3 Axial coding: thematic cluster induction

Based on the hierarchical nodes, the study further integrated the main categories and identified the logical relationships between them, forming an axial coding structure with perception-emotion-understanding-background as the main line. This structure reveals the dynamic path of the audience in the process of understanding the artwork, and also reflects the differences between different groups (see Table 3).

**Table 3:** Comparison of citation frequency between the two groups of students at the topic cluster level (unit: number of citations).

Theme cluster	Child node example	Arts	Non-art
Visual perception pathway	Color attention, shape/line, composition/detail observation	140	114
Abstract reasoning comprehension	Symbolic interpretation, comprehension barriers, academic narrative, and subjective speculation	97	54
Intuitive recognition	Artistic identification, narrative construction, and life-related associations	68	44
Emotionally driven reactions	Emotional associations, positive/negative/mixed emotions	39	42
Background impact factor	Subject background and personal experience	58	35

Both groups of students prioritized visual perception, indicating that sensory perception is a common starting point for artistic understanding. Artistic students excelled in deep understanding and contextual application, demonstrating strong abstract reasoning and knowledge integration abilities. Non-artistic students, on the other hand, tended to rely more on perceptual and fragmented speculation. Artistic students tended to organize visual information into coherent narratives, while non-artistic students relied more on emotional responses and personal experience. Overall, artistic students exhibited a deeper and more structured understanding, while non-artistic students exhibited a more perceptual and experiential understanding.

### 4.4 Selective coding: understanding the path model

After clustering the thematic clusters, this study conducted further selective coding to integrate the relationships between the different thematic clusters and construct a model of the understanding pathways of abstract and figurative art. This stage of analysis focused on identifying the core pathways of students' art reception and revealing the differences between different groups.

#### 4.4.1 Selective coding table

Table 4 shows that both groups of students began their visual perception of artwork, but their paths to understanding diverged significantly. Art students, drawing on their professional background, were able to engage in abstract reasoning or symbolic interpretation after perceiving artwork. When it came to figurative art, they achieved systematic understanding through narrative construction. Non-art students, however, relied more heavily on emotion and experience, prone to comprehension difficulties or subjective speculation when faced with abstract art. When it came to figurative art, they generated meaning through emotional and everyday associations. Overall, art students' understanding was more structured and in-depth, while non-art students were more emotionally and individually engaged.

**Table 4:** Selective coding results of abstract and figurative art.

Types of art	Group	Main path	Branching path
Abstract art	Art students	Visual perception → emotional drive → contextual factors → abstract reasoning → symbolic interpretation	Background influence → artistic identification
	Non-arts students	Visual perception → emotional drive → contextual factors → comprehension barriers	Background influence → subjective speculation
Figurative art	Art students	Visual perception → intuitive recognition → background factors → narrative construction → academic narrative	Background factors → art recognition
	Non-arts students	Visual perception → intuitive recognition → background factors → emotional drive → life-related association	

#### 4.4.2 Understanding the path model

**Figure 1:**
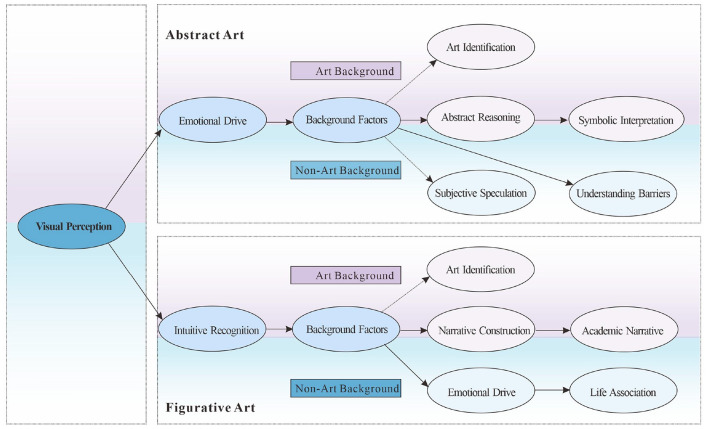
Artwork comprehension path model.

Based on the results of selective coding, this study constructed a “Pathway Model for Artwork Comprehension.” This model contrasts typical pathways for abstract and figurative art and identifies differences between art and non-art students across different types of artwork (see Figure 1).

Based on the results of selective coding, this study constructed a “path model for understanding artworks” to compare the typical paths of abstract and figurative art in two groups. The results showed that regardless of the group or type, understanding starts from visual perception, confirming the “holistic perception” of the Gestalt model and the “perceptual analysis” stage of the Leder model. In terms of path differentiation, art students in abstract art often go through the professional path of “emotion → background → reasoning → symbolic interpretation,” while non-art students stay at the experiential understanding of “background → understanding barriers/subjective speculation”; in figurative art, the former develops along the path of “intuition → background → narrative → academic,” while the latter relies on “intuition → background → emotion → association,” and the understanding is more emotional. Background factors play a key regulatory role here and are the core condition for deepening understanding.

In general, art understanding is a social construction process influenced by background and cultural experience. This model integrates psychology, semiotics and aesthetics perspectives to provide systematic empirical evidence for the differences in understanding of abstract and figurative art.

## 5 Quantitative research

### 5.1 Research questions and hypotheses

The quantitative part of this study is based on the “Art Understanding Path Model” constructed in the qualitative phase, which distinguishes between abstract and figurative art and compares the typical paths of art and non-art students (see Figure 1). The model shows that both groups start with visual perception, but their subsequent paths diverge significantly, and background factors play a core role in multiple paths. The study focuses on three points: (1) The differences between the two groups in abstract art between “emotion-background-reasoning/symbolization” and “background-obstacle/speculation.” (2) The differential structure of “intuition-background-narrative/academic” and “intuition-background-emotion-association” in figurative art. (3) The mediating and moderating role of background factors.

Based on this hypothesis:

H1 (Abstract/Art): There is a main path of “perception → emotion → background → abstract reasoning → symbolic interpretation” and a branch of “background → art recognition.”
H1a (Abstract/Non-artistic): There is a main path of “perception → emotion → background → comprehension barrier” and a branch of “background → subjective inference.”
H2 (Figurative/Artistic): There is a main path of “intuitive → background → narrative → academic” and a branch of “background → art identification.”
H2a (Figurative/non-artistic): There is a main path of “intuition → background → emotion → life association.”
H3 (Overall): Artistic pathways are more systematic/professional, non-artistic pathways are more experiential/emotional, and contextual factors are the key hub for deepening understanding.

### 5.2 Research model construction

Based on the qualitative results, this study constructs a model of art understanding pathways starting from visual perception, depicting the typical pathways and branches of art and non-art students in abstract and figurative art, revealing the dynamic relationship between perception, emotion, context and reasoning, and the moderating role of educational background.

Path test results show that the abstract-artistic category follows a main chain of “perception → emotion → context → abstract reasoning → symbolic interpretation,” with a branch of “context → art identification.” The non-artistic category follows a chain of “perception → emotion → context → comprehension impairment/subjective speculation.” The figurative-artistic category follows a chain of “intuition → context → narrative → academic” and “context → art identification,” while the non-artistic category follows a chain of “intuition → context → emotion → everyday association,” reflecting differences in structure and experience between groups.

### 5.3 Data analysis

#### 5.3.1 Descriptive statistics

Based on the analysis results, the mean scores for each core dimension ranged from 3.15 to 3.78, indicating widespread agreement among participants with the relevant statements. Visual perception scored highest, indicating that sensory perception and formal recognition are common starting points for artistic comprehension. Symbolic interpretation, abstract reasoning, and narrative construction within the comprehension dimension were above average, reflecting a certain tendency toward meaning construction. The dispersion of emotional drive and life-related associations was relatively high, indicating significant individual differences in emotional response and experiential understanding. In the abstract work condition, visual perception and intuitive recognition remained prominent, while the dispersion of emotional and life-related associations increased further. (See Supplementary Appendix D for basic descriptive statistical indicators).

#### 5.3.2 Reliability analysis

To test the internal consistency of the scale, Cronbach's α, CITC, and “deleted α” were used. The overall standardized α was 0.964, indicating excellent reliability (see Table 5). The CITC for each construct was mostly ≥0.60, indicating good homogeneity and discrimination. The CITC for contextual factors, abstract reasoning, symbolic interpretation, narrative construction, and academic narrative were all in the range of 0.77–0.84 (deleted α ≈ 0.961). The CITC for comprehension barriers was slightly lower at 0.340 (deleted α = 0.966), but was retained as it reflects “impeded comprehension experiences.” Under the abstract (x) condition, reliability was consistent with the main scale, with CITCs for the main dimensions ranging from 0.74 to 0.81 (α ≈ 0.961–0.962). Only the CITC for x comprehension barriers was slightly lower (0.434, α = 0.965), indicating good overall reliability.

**Table 5:** Reliability analysis table.

Number of items	Sample size	α coefficient
22	665	0.964

#### 5.3.3 Validity analysis

The measurement model included eight latent variables (perception, emotion, understanding, context, and their corresponding x-dimensions), a total of 28 observational indicators, and a sample size of 665. Perception and context each contained two indicators, emotion three items, and understanding encompassed seven sub-dimensions (such as abstract reasoning, symbolic interpretation, narrative construction, and everyday associations). The x-dimension structure was consistent with the main model, providing a solid foundation for CFA and multi-group comparisons.

The loading results (see Table 6) show that the “professional understanding” item has a high and significant loading, with good convergent validity; the “understanding barriers” and other barrier/experience understanding items have relatively low loadings, but reflect the uniqueness of negative experiences and do not affect theoretical validity, indicating that the two types of understanding paths are distinguishable in terms of psychological connotations.

**Table 6:** Factor loading coefficient table.

Factor (latent variable)	Measurement item (manifest variable)	Non-standard load factor (coef.)	Std. error	*z* (CR value)	*P*-value	Standard load factor (std. estimate)	SMC	AVE	CR
Perception	Intuitive identification	1.000	—	—	—	0.812	0.659	0.622	0.767
Perception	Visual perception	0.730	0.034	21.356	0.000	0.765	0.585		
Emotion	Mixed emotions	1.000	—	—	—	0.757	0.573	0.446	0.705
Emotion	Negative emotions	0.985	0.063	15.758	0.000	0.614	0.377		
Emotion	Positive emotions	0.786	0.049	15.969	0.000	0.622	0.386		
Understand	Academic narrative	1.000	—	—	—	0.778	0.605	0.598	0.907
Understand	Artistic identification	0.950	0.043	22.084	0.000	0.781	0.609		
Understand	Comprehension barriers	0.361	0.048	7.528	0.000	0.294	0.086		
Understand	Symbolic interpretation	1.049	0.041	25.364	0.000	0.869	0.755		
Understand	Abstract reasoning	1.143	0.043	26.507	0.000	0.898	0.806		
Understand	Narrative construction	1.139	0.044	25.628	0.000	0.876	0.767		
understand	Lifestyle association	1.014	0.049	20.850	0.000	0.745	0.555		
Background	Personal experience	1.000	—	—	—	0.807	0.652	0.676	0.807
Background	Discipline background	1.198	0.047	25.465	0.000	0.837	0.701		
x Perception	x Intuitive recognition	1.000	—	—	—	0.890	0.792	0.696	0.820
x Perception	x Visual Perception	0.760	0.033	22.866	0.000	0.775	0.600		
x Emotion	x Complex emotions	1.000	—	—	—	0.911	0.830	0.552	0.778
x Emotion	x Negative emotions	0.942	0.043	22.021	0.000	0.750	0.563		
x Emotion	x Positive emotions	0.490	0.036	13.606	0.000	0.513	0.263		
x Understanding	x Abstract reasoning	1.000	—	—	—	0.865	0.749	0.619	0.917
x Understanding	x Subjective speculation	1.040	0.034	30.842	0.000	0.870	0.758		
x Understanding	x Art recognition	0.946	0.035	26.785	0.000	0.806	0.650		
x Understanding	x Lifestyle association	0.960	0.037	25.661	0.000	0.786	0.618		
x Understanding	x Academic narrative	0.985	0.036	26.981	0.000	0.810	0.655		
x Understanding	x Comprehension disorder	0.541	0.047	11.455	0.000	0.427	0.182		
x Understanding	Symbolic interpretation of x	0.987	0.034	29.450	0.000	0.850	0.722		
x Background	x Personal experience	1.000	—	—	—	0.820	0.673	0.698	0.822
x Background	x Subject background	1.180	0.045	26.279	0.000	0.851	0.724		

In terms of convergent validity, most constructs had CR ≥ 0.70 and AVE ≥ 0.50, or were close to the threshold, such as perception (0.622/0.767), understanding (0.598/0.907), and context (0.676/0.807). Under the concrete condition, xperception (0.696/0.820), xcomprehension (0.619/0.917), and xcontext (0.698/0.822) all performed well. Only emotion showed a satisfactory CR but a slightly lower AVE (0.446/0.705). Within the dual-domain caliber, its proximity to “blocked/experiential understanding” can be interpreted as a difference in understanding style rather than a measurement confound.

Table 7 shows that the AVE square root value of each latent variable is higher than its correlation coefficient with other latent variables, indicating that the model has good discriminant validity. Among them, the correlations between understanding and perception, and between background and extended background are relatively high, but still do not exceed their respective AVE square root values. This indicates that although there are certain connections between the latent variables, they can still be effectively distinguished, and the measurement model has good discriminant validity.

**Table 7:** Discriminant validity: pearson correlation and AVE square root value.

Construct	Perception	Emotion	Understand	Background	x Perception	x Emotion	x Understanding	x Background
Perception	**0.789**							
Emotion	0.643	**0.668**						
Understand	0.752	0.753	**0.773**					
Background	0.681	0.598	0.707	**0.822**				
x Perception	0.690	0.511	0.697	0.684	**0.834**			
x Emotion	0.550	0.708	0.701	0.617	0.597	**0.743**		
x Understanding	0.655	0.646	0.704	0.760	0.679	0.767	**0.787**	
x Background	0.617	0.534	0.721	0.794	0.671	0.598	0.732	**0.836**

The bold numbers on the diagonal line are the square root values of AVE.

The overall fit index is influenced by the complex structure of “cross-contextual juxtaposition + multidimensional understanding.” Within this explanatory framework, measurement rationality and structural simplicity should be the primary considerations. The paths and covariances between professional understanding and other constructs provide clear theoretical guidance. This allows residuals and value-added indicators, even when they fall short of ideal thresholds, to be interpreted as acceptable or borderline acceptable. Subsequent path tests within the structural model for the four scenarios further validated the explanatory power of this distinction (see Table 8 for details).

**Table 8:** Model fitting indicators.

Common indicators	χ^2^	df	*P*-value	Chi-square degrees of freedom ratio χ^2^/df	GFI	RMSEA	RMR	CFI	NFI	NNFI
Judgment criteria	—	—	>0.05	<3	>0.9	<0.10	<0.05	>0.9	>0.9	>0.9
Value	3672.181	322	0.000	11.404	0.685	0.125	0.067	0.799	0.784	0.764
Other indicators	TLI	AGFI	IFI	PGFI	PNFI	PCFI	SRMR	RMSEA 90%CI		
Judgment criteria	>0.9	>0.9	>0.9	>0.5	>0.5	>0.5	<0.1	—		
Value	0.764	0.603	0.799	0.543	0.668	0.680	0.096	0.112–0.131		

Default model $\chi_{\left(378\right)}^{2}$ = 17,010.570, *p* = 1.000; AIC = 156.956, BIC = 534.938.

Standardized covariances (approximate latent correlations) revealed core connections: perception-understanding 0.891, perception-context 0.863, and perception-emotion 0.855 (all *p* < 0.001; see Table 9). Under the concrete (x) caliber, the “professional understanding-context” connection was stronger, reflecting the support provided by context for deepening understanding. The “blocked/experiential understanding-emotion” connection was stronger, reflecting that under concrete conditions, emotional/experiential understanding pathways were more likely to be accompanied by “blockage.”

**Table 9:** Factor covariance table.

Factor	Factor	Unstandardized estimated coefficients (coef.)	Standard error (std. error)	*z*	*P*-value	Standardized estimated coefficients (std. estimate)
Perception	Emotion	0.362	0.029	12.695	0.000	0.855
Perception	Understand	0.409	0.030	13.541	0.000	0.891
Perception	Background	0.363	0.027	13.347	0.000	0.863
Perception	x Perception	0.419	0.030	13.854	0.000	0.857
Perception	x Emotion	0.336	0.030	11.139	0.000	0.610
Perception	x Understanding	0.376	0.029	13.164	0.000	0.778
Perception	x Background	0.338	0.027	12.600	0.000	0.775
Emotion	Understand	0.363	0.028	12.923	0.000	0.869
Emotion	Background	0.303	0.025	12.204	0.000	0.791
Emotion	x Perception	0.288	0.026	11.047	0.000	0.646
Emotion	x Emotion	0.435	0.032	13.577	0.000	0.866
Emotion	x Understanding	0.334	0.027	12.470	0.000	0.757
Emotion	x Background	0.278	0.024	11.383	0.000	0.701
Understand	Background	0.387	0.028	13.867	0.000	0.931
Understand	x Perception	0.377	0.028	13.222	0.000	0.780
Understand	x Emotion	0.362	0.030	12.189	0.000	0.665
Understand	x Understanding	0.423	0.030	14.071	0.000	0.885
Understand	x Background	0.353	0.027	13.154	0.000	0.821
Background	x Perception	0.370	0.027	13.747	0.000	0.837
Background	x Emotion	0.334	0.028	12.066	0.000	0.670
Background	x Understanding	0.383	0.027	14.142	0.000	0.876
Background	x Background	0.384	0.027	14.413	0.000	0.974
x Perception	x Emotion	0.338	0.030	11.293	0.000	0.583
x Perception	x Understanding	0.404	0.029	13.987	0.000	0.794
x Perception	x Background	0.373	0.027	13.628	0.000	0.814
x Emotion	x Understanding	0.426	0.031	13.597	0.000	0.742
x Emotion	x Background	0.323	0.028	11.572	0.000	0.625
x Understanding	x Background	0.419	0.029	14.628	0.000	0.923

Overall, the measurement tool in this study demonstrated good convergent validity. The CR and AVE of most constructs met or approached the standards. The loadings on each dimension of professional understanding (e.g., abstract reasoning, symbolic interpretation, and narrative construction) were significant, reflecting positive progress in understanding. Although some indicators within the “blocked experiential understanding” dimension (e.g., “understanding barriers”) exhibited low loadings, they remain theoretically significant, reflecting distinct stages of understanding barriers.

In terms of discriminant validity, according to the Fornell–Larcker criterion, the AVE of most constructs was higher than the correlation coefficient, indicating good measurement discrimination. The high correlations between emotion, understanding, and context are consistent with theoretical proximity, indicating different paths to understanding. While the overall fit index was slightly below the ideal threshold, it remained within an acceptable range considering the complexity of the model. The measurement tool is generally robust and can support subsequent path and multi-group analyses.

#### 5.3.4 Structural model and path test

##### 5.3.4.1 Abstract—Art

Table 10 shows that the main path “vision → emotion → background → abstract reasoning → symbolic interpretation” and the branch “background → art recognition” were both significant (β = 0.546–0.842, *p* < 0.001), indicating that perception is activated by emotion and background, gradually leading to higher-order understanding, presenting a specialized path of “perception-background-abstraction.”

**Table 10:** Summary of regression coefficients of abstract-art model.

*X*	→	*Y*	Unstandardized path coefficients	*SE*	*z* (CR value)	*P*-value	Standardized path coefficient
Emotionally driven	→	Background factors	0.668	0.035	19.247	0.000	0.598
Background factors	→	Artistic Identification	0.699	0.032	21.645	0.000	0.643
Visual perception	→	Emotionally driven	0.566	0.034	16.786	0.000	0.546
Background factors	→	Abstract reasoning	0.823	0.030	27.029	0.000	0.724
Abstract reasoning	→	Symbolic interpretation	0.798	0.020	40.201	0.000	0.842

→ indicates path influence relationship.

Table 11, model fit indices showed GFI = 0.790, CFI = 0.822, NFI = 0.819, RMR = 0.102, and RMSEA = 0.268 (90% CI ≈ 0.25–0.29). Although the absolute fit indices did not reach the ideal threshold, incremental fit (such as CFI) approached the acceptable limit. With large sample sizes and a large number of pathways and latent variables, it is common for chi-square correlation indicators to be somewhat sensitive. This study focused on the theoretical consistency and robustness of pathway directions and effect sizes.

**Table 11:** Abstract-art model fitting index.

Common indicators	χ^2^	df	*P*-value	Chi-square degrees of freedom ratio χ^2^/df	GFI	RMSEA	RMR	CFI	NFI	NNFI
Judgment criteria	—	—	>0.05	<3	>0.9	<0.10	<0.05	>0.9	>0.9	>0.9
Value	485.863	10	0.000	48.586	0.790	0.268	0.102	0.822	0.819	0.732
Other indicators	TLI	AGFI	IFI	PGFI	PNFI	PCFI	SRMR	RMSEA 90%CI		
Judgment criteria	>0.9	>0.9	>0.9	>0.5	>0.5	>0.5	<0.1	—		
Value	0.732	0.560	0.822	0.376	0.546	0.548	0.181	0.248–0.288		

Default model $\chi_{\left(15\right)}^{2}$ = 2,682.076, *p* = 1.000; AIC = 20.539, BIC = 70.036.

Table 12 shows the model fit *R*^2^, which shows the explanatory power of each key outcome variable: abstract reasoning *R*^2^ = 0.523, symbolic interpretation *R*^2^ = 0.708, and art identification *R*^2^ = 0.413, indicating that the model has a strong explanatory power for “deep understanding” and an above-average explanatory power for “professional identification/naming.”

**Table 12:** Summary of the *R*^2^ of the abstract-art model fit.

Item	*R*-squared value
Background factors	0.358
Artistic Identification	0.413
Emotionally driven	0.298
Abstract reasoning	0.523
Symbolic interpretation	0.708

**Figure 2:**
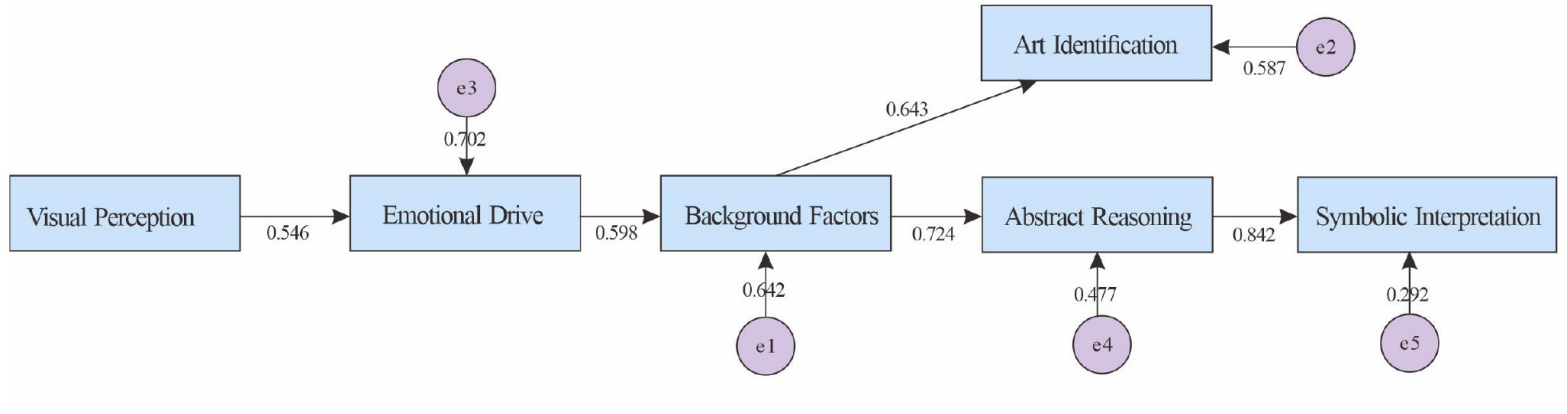
Validation results of the abstract-artistic understanding model.

In Figure 2 presents the main chain of “perception-emotion-background-abstraction-symbolization” in accordance with the above numerical values, and takes “background → art recognition” as a parallel branch, intuitively strengthening the key mediating role of “background factors” in deepening abstract understanding.

##### 5.3.4.2 Abstract—Non-art

Table 13 summarizes the model regression coefficients, supporting the empirical pathways of “visual perception → emotional drive → background factors → comprehension barriers” and “background factors → subjective inference”: visual perception positively stimulates emotional drive (β = 0.546, *p* < 0.001), which in turn enhances background factors (β = 0.598, *p* < 0.001). Background factors significantly predict comprehension barriers (β = 0.270, *p* < 0.001) and promote subjective inference (β = 0.731, *p* < 0.001). This structure suggests that non-artistic individuals are more likely to remain in empirical interpretations or experience blockages after the “emotion-background” equation in abstract situations.

**Table 13:** Summary of regression coefficients of abstract-non-artistic model.

*X*	→	*Y*	Unstandardized path coefficients	*SE*	*z* (CR value)	*P*-value	Standardized path coefficient
Background factors	→	Comprehension barriers	0.296	0.041	7.229	0.000	0.270
Emotionally driven	→	Background factors	0.667	0.035	19.239	0.000	0.598
Background factors	→	Subjective speculation	0.849	0.031	27.619	0.000	0.731
Visual perception	→	Emotionally driven	0.567	0.034	16.792	0.000	0.546

→ indicates path influence relationship.

In Table 14 show a GFI of 0.832, a CFI of 0.775, a NFI of 0.772, a RMR of 0.078, and a RMSEA of 0.281 (90% CI ≈ 0.26–0.31). Similar to the Abstract/Art category, the chi-square/degrees of freedom ratio is high in complex models and with large sample sizes; however, the key paths all reach significance and have effect sizes above medium, reflecting the “empiricism-blocked/speculated” understanding pattern well.

**Table 14:** Abstract-non-art model fitting indicators.

Common indicators	χ^2^	df	*P*-value	Chi-square degrees of freedom ratio χ2/df	GFI	RMSEA	RMR	CFI	NFI	NNFI
Judgment criteria	—	—	>0.05	<3	>0.9	<0.10	<0.05	>0.9	>0.9	>0.9
Value	321.411	6	0.000	53.569	0.832	0.281	0.078	0.775	0.772	0.624
Other indicators	TLI	AGFI	IFI	PGFI	PNFI	PCFI	SRMR	RMSEA 90%CI		
Judgment criteria	>0.9	>0.9	>0.9	>0.5	>0.5	>0.5	<0.1	—		
Value	0.624	0.581	0.775	0.333	0.463	0.465	0.142	0.255–0.308		

Default model $\chi_{\left(10\right)}^{2}$ = 1,409.498, *p* = 1.000; AIC = 17.033, BIC = 57.531.

In Table 15 shows that subjective speculation *R*^2^ = 0.534, background factors *R*^2^ = 0.358, emotional drive *R*^2^ = 0.298, and comprehension barrier *R*^2^ = 0.073, indicating that the model has a high degree of explanation for “subjective speculation” and a limited explanation for the variation in “comprehension barrier,” suggesting that blockage may also be affected by other factors not included (such as task requirements, time pressure, etc.), which is consistent with the understanding that “blockage” is a multi-source phenomenon.

**Table 15:** Summary of the *R*^2^ fit of the abstract-non-artistic model.

Item	*R*-squared value
Comprehension barriers	0.073
Background factors	0.358
Subjective speculation	0.534
Emotionally driven	0.298

**Figure 3:**
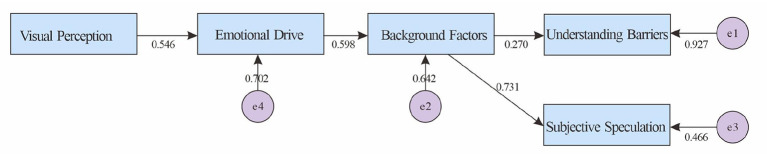
Validation results of the abstract-non-artistic understanding model.

Figure 3 shows the abstract-non-art model diagram, which vividly presents the two branches mentioned above: one leading to “understanding barriers” and the other leading to “subjective speculation,” which together depict the characteristics of non-artistic categories that rely more on emotions/backgrounds in front of abstract works but find it more difficult to transition to abstract and symbolic interpretations.

##### 5.3.4.3 Figurative art

In Table 16 reveal a scholarly path of “intuitive identification—background factors—narrative construction—academic narrative,” with a branching relationship from “background factors → artistic identification”: x visual perception → x intuitive identification (β = 0.689, *p* < 0.001), x intuitive identification → x background factors (β = 0.658, *p* < 0.001), x background factors → x narrative construction (β = 0.716, *p* < 0.001), and x narrative construction → x academic narrative (β = 0.654, *p* < 0.001). Furthermore, the branching relationship from x background factors to x artistic identification was also significant (β = 0.692, *p* < 0.001). This chain demonstrates a clear progression from intuitive representation to professional narrative/academic expression.

**Table 16:** Summary of regression coefficients of the figurative-artistic model.

*X*	→	*Y*	Unstandardized path coefficients	*SE*	*z* (CR value)	*P*-value	Standardized path coefficient
x Visual perception	→	x Intuitive recognition	0.789	0.032	24.530	0.000	0.689
x Narrative construction	→	x Academic narrative	0.665	0.030	22.273	0.000	0.654
x Intuitive recognition	→	x Background factors	0.628	0.028	22.559	0.000	0.658
x Background factors	→	x Art recognition	0.750	0.030	24.720	0.000	0.692
x Background factors	→	x Narrative construction	0.790	0.030	26.436	0.000	0.716

→ indicates path influence relationship.

In Table 17 show a GFI of 0.775, a CFI of 0.765, a NFI of 0.763, an RMR of 0.112, and an RMSEA of 0.310 (90% CI ≈ 0.29–0.33). Although the absolute fit is somewhat tight, the core path effect sizes are stable and consistent in direction. This, combined with the convergence/discrimination evidence at the measurement level, supports the interpretation of the structural results.

**Table 17:** Fitting index of the figurative-artistic model.

Common indicators	χ^2^	df	*P*-value	Chi-square degrees of freedom ratio χ^2^/df	GFI	RMSEA	RMR	CFI	NFI	NNFI
Judgment criteria	—	—	>0.05	<3	>0.9	<0.10	<0.05	>0.9	>0.9	>0.9
Value	649.451	10	0.000	64.945	0.775	0.310	0.112	0.765	0.763	0.648
Other indicators	TLI	AGFI	IFI	PGFI	PNFI	PCFI	SRMR	RMSEA 90%CI		
Judgment criteria	>0.9	>0.9	>0.9	>0.5	>0.5	>0.5	<0.1	—		
Value	0.648	0.527	0.766	0.369	0.509	0.510	0.169	0.290–0.331		

Default model $\chi_{\left(15\right)}^{2}$ = 2,737.846, *p* = 1.000; AIC = 20.047, BIC = 69.544.

Table 18, xnarrative construction *R*^2^ = 0.512, xartistic identification *R*^2^ = 0.479, xintuitive identification *R*^2^ = 0.475, xbackground factors *R*^2^ = 0.434, and xacademic narrative *R*^2^ = 0.427, indicating that the chain from intuition to narrative/academic has a stable explanatory power.

**Table 18:** Summary of *R*^2^ fit of the figurative-artistic model.

Item	*R*-squared value
x Intuitive recognition	0.475
x Academic narrative	0.427
x Background factors	0.434
x Art recognition	0.479
x Narrative construction	0.512

**Figure 4:**
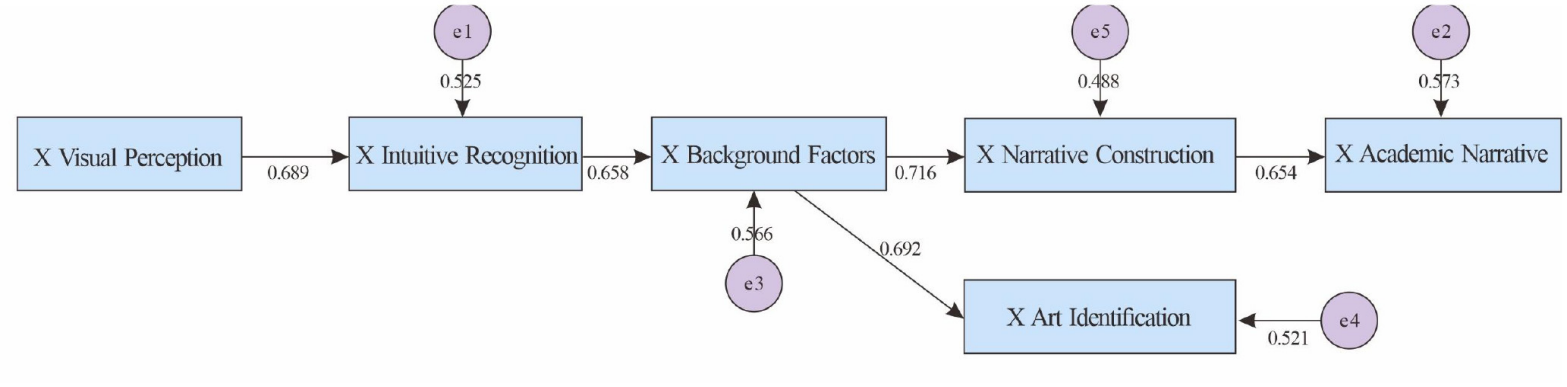
Verification results of the figurative-artistic understanding model.

Figure 4 is consistent with the path table, highlighting the pivotal role of “background factors”: it not only leads to the deep line of “narrative construction-academic narrative,” but also strengthens “artistic identification” in parallel.

##### 5.3.4.4 Figurative—Non-artistic

The results in Table 19 support the empirical path of “intuitive identification → context → emotion → life-related association”: all path coefficients are significant (β = 0.689, 0.658, 0.598, 0.692, *p* < 0.001). This suggests that non-artistic people primarily construct understanding through emotion and daily experience in concrete contexts, rather than engaging in academic research.

**Table 19:** Summary of regression coefficients of the figurative-non-artistic model.

*X*	→	*Y*	Unstandardized path coefficients	*SE*	*z* (CR value)	*P*-value	Standardized path coefficient
x Visual perception	→	x Intuitive recognition	0.789	0.032	24.535	0.000	0.689
x Background factors	→	x Emotional drive	0.560	0.029	19.224	0.000	0.598
x Intuitive recognition	→	x Background factors	0.628	0.028	22.562	0.000	0.658
x Emotional drive	→	x Lifestyle association	0.832	0.034	24.695	0.000	0.692

→ indicates path influence relationship.

In Table 20 show a GFI of 0.876, a CFI of 0.865, a NFI of 0.862, an RMR of 0.086, and an RMSEA of 0.245 (90% CI ≈ 0.22–0.27). Compared to other scenarios, this model has a better value-added fit, indicating that the structure of “intuition-context-emotion-life association” is more consistent and stable in the context of non-artistic, figurative works.

**Table 20:** Fitting index of the figurative-non-artistic model.

Common indicators	χ^2^	df	*P*-value	Chi-square degrees of freedom ratio χ^2^/df	GFI	RMSEA	RMR	CFI	NFI	NNFI
Judgment criteria	—	—	>0.05	<3	>0.9	<0.10	<0.05	>0.9	>0.9	>0.9
Value	245.382	6	0.000	40.897	0.876	0.245	0.086	0.865	0.862	0.774
Other indicators	TLI	AGFI	IFI	PGFI	PNFI	PCFI	SRMR	RMSEA 90%CI		
Judgment criteria	>0.9	>0.9	>0.9	>0.5	>0.5	>0.5	<0.1	—		
Value	0.774	0.689	0.865	0.350	0.517	0.519	0.140	0.219–0.272		

Default model $\chi_{\left(10\right)}^{2}$ = 1,778.707, *p* = 1.00; AIC = 17.262, BIC = 57.760.

In Table 21 shows that the xnarrative/association-related outcome variables all have moderate explanatory power: xnarrative construction *R*^2^ = 0.512, xlife-related association *R*^2^ = 0.478, xintuitive identification *R*^2^ = 0.475, xbackground factor *R*^2^ = 0.434, xemotional drive *R*^2^ = 0.357, which is consistent with the chain progression of “from intuition-background-emotion to daily association.”

**Table 21:** Summary of *R*^2^ fit of the figurative-non-artistic model.

Item	*R*-squared value
x Intuitive recognition	0.475
x Emotional drive	0.357
x Background factors	0.434
x Lifestyle association	0.478

**Figure 5:**
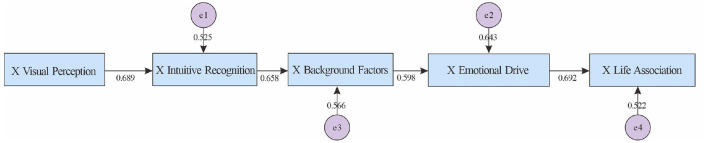
Verification results of the figurative-non-artistic understanding model.

Figure 5 shows the figurative-non-artistic model diagram, which intuitively presents the endpoint characteristics of “empirical association” and further supports the consistency across methods (qualitative → quantitative).

### 5.4 Results

#### 5.4.1 Hypothesis test results

H1a (Abstract-Art): The paths “Visual Perception → Affective Motivation → Context → Abstract Reasoning → Symbolic Interpretation” were all significant, with increasing effects (β Emotion → Context ≈ 0.60; β Context → Reasoning ≈ 0.72; β Reasoning → Symbolic Interpretation ≈ 0.84). The significant branch “Context → Artistic Identification” (β ≈ 0.64) remained. The endpoint explanatory power was high (*R*^2^ Symbol ≈ 0.71), confirming the path of deepening specialization and confirming the hypothesis.

H1b (Abstract-Non-Art): The first segment, “Vision → Emotion → Context,” was significant, with Context split between “Comprehension Impediment” (β ≈ 0.27) and “Subjective Inference” (β ≈ 0.73), with the latter having a higher explanatory power (*R*^2^ ≈ 0.53). The results indicate that non-artistic individuals tend to favor empirical interpretations and experience impairment in abstract comprehension, supporting the hypothesis.

H2a (Figurative-Artistic): The main chain “Intuitive Identification → Background → Narrative → Scholarship” was significant (β Background → Narrative ≈ 0.72; β Narrative → Scholarship ≈ 0.65), and “Background → Artistic Identification” (β ≈ 0.69) was retained. Explanatory power was moderate to high (*R*^2^ ≈ 0.43–0.51), demonstrating a hierarchical path from intuitive to scholarly, supporting the hypothesis.

H2b (Figurative-non-artistic): The path “intuitive identification → background → emotion → life-related association” is significant, with the strongest effect at the end (β ≈ 0.69, *R*^2^ ≈ 0.48), indicating that non-artistic categories use emotion and experience to complete figurative interpretation, and the hypothesis is established.

H3 (Overall Difference): Artistic subjects have a stronger link from “background → abstract reasoning/narrative → symbolization/academic narrative,” while non-artistic subjects have a stronger link from “background → subjective speculation/emotion → everyday association.” These four models collectively confirm the pivotal role of context in deepening and diverting understanding, and the hypothesis generally holds.

#### 5.4.2 Model results and corrections

**Figure 6:**
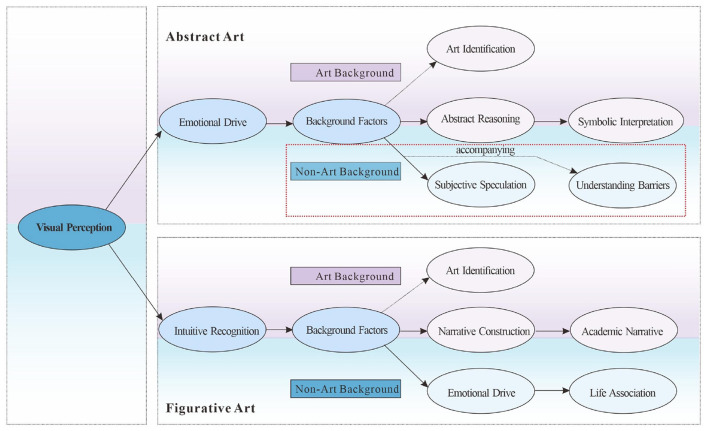
Final understanding path model.

The discriminant validity of the constructs was assessed using MSV and ASV, as shown in Table 22. Only significant paths were retained from the four revised models, and standardized coefficients and *R*^2^ were annotated. The final understanding of the path model is shown in Figure 6.

**Table 22:** Discriminant validity indicators MSV and ASV.

Item	AVE value	CR combined reliability value	MSV value	ASV value
Perception	0.622	0.767	0.793	0.611
Emotion	0.446	0.705	0.754	0.530
Understand	0.598	0.907	0.867	0.685
Background	0.676	0.807	0.949	0.675
x Perception	0.696	0.820	0.734	0.585
x Emotion	0.552	0.778	0.750	0.471
x Understanding	0.619	0.917	0.853	0.681
x Background	0.698	0.822	0.949	0.660

MSV value, maximum of shared squared variance; ASV value, average of shared squared variance.

Figure 2 (Abstract-Art) shows the main chain as “visual perception → emotion-driven → contextual factors → abstract reasoning → symbolic interpretation,” with the “context → art recognition” branch retained. The “abstract reasoning → symbolic interpretation” effect was the strongest (β ≈ 0.842) and had the highest explanatory power (*R*^2^ ≈ 0.708), demonstrating a specialized path from perception/emotion through contextual support to higher-level understanding.

Figure 3 (abstract-non-artistic) shows the main line of “visual perception → emotional drive → background factors,” and the background is further divided into “understanding barriers” (β ≈ 0.270) and “subjective speculation” (β ≈ 0.731). The latter has a higher explanatory power (*R*^2^ ≈ 0.534), indicating that non-artistic people are more inclined to experiential interpretation in abstract situations and are accompanied by understanding barriers.

The model in Figure 4 (Figurative-Art) is “Intuitive Identification → Background Factors → Narrative Construction → Academic Narrative,” with a branch path from “Background → Artistic Identification.” The key paths are robust (β background → narrative ≈ 0.716; β narrative → academic ≈ 0.654), and the explanatory power of each outcome variable is moderate (*R*^2^ ≈ 0.43–0.51), reflecting a deepening of narrative from intuitive to academic.

Figure 5 (Figurative-non-artistic) retains the path of “intuitive recognition → background factors → emotional drive → life-related association,” and the final segment effect is significant (β≈0.692, *R*^2^≈0.478), indicating that non-artistic people mainly complete their understanding of figurative works through the integration of emotion and experience.

## 6 Discussions

### 6.1 Research findings and path differences

Based on the pathway evidence presented in Chapter 4, the four-quadrant model suggests that “visual perception” is the common starting point for both groups of participants, while contextual factors determine subsequent pathways. When contextual resources (disciplinary knowledge, artistic training, and relevant experience) are effectively mobilized, comprehension is more likely to progress toward abstract reasoning/symbolic interpretation or narrative construction/academic narrative. When context is insufficient or mismatched, the pathway diverges toward experiential interpretation (subjective speculation, everyday associations), leading to even comprehension barriers. This finding aligns with the “perception-interpretation” sequence in the information-processing aesthetics framework (Leder et al., 2004; Leder and Nadal, 2014), but further elevates “context” from a contextual auxiliary variable to a pivotal factor that serves as both a mediator (undertaking perception/emotion and promoting higher-level comprehension) and a moderator (altering the strength and direction of the pathway across different groups/artwork types). This also echoes the perspective of reception aesthetics on

the “structure of expectation/history of effect,” which states that knowledge and experiential frameworks shape the boundaries and depth of understanding (Jauss, 1982; Gadamer, 1989; Tribot et al., 2024; Bara et al., 2025). Emotion here is neither a unidirectional facilitator nor an obstacle, but rather a conditional element that, in different contexts, co-reconstructs the pathway alongside cognition (Pelowski et al., 2017; Vartanian and Chatterjee, 2021; Díaz-Vera, 2025). Among the four contexts, art students' understanding pathways are more hierarchical. In abstract art, they often progress through perception, emotion, and context, leading to abstract reasoning and symbolic interpretation, while in figurative art, they develop narratives through intuitive recognition and contextual support. Non-art students rely more on emotion and experience, becoming easily blocked in abstract contexts and converging on emotional associations for experiential understanding in figurative contexts. Quantitative analysis confirms the mediating role of context in the “perception/emotion → higher-order understanding” transition, with significant differences observed across groups and artwork types. The results show that perception is the common starting point, and context-emotion coupling determines the level of understanding, reflecting the systematic differentiation of artistic and experiential types.

### 6.2 Theoretical contributions and practical significance

On a theoretical level, this study proposes a “hub-divergence mechanism of context,” revealing its dual role in artistic comprehension: it serves as a mediator connecting the “perception-emotion-comprehension” chain, supporting the construction of meaning, while also exhibiting directional divergence across groups and work types. When the context is sufficient and appropriate, audiences achieve a deeper understanding along the path of “perception-emotion-context-reasoning-symbolic interpretation.” Conversely, they shift to experiential understanding through “perception-emotion-experience-association.” This mechanism transcends the previous limitation of viewing context as a passive variable, elevating it to a cognitive hub of active regulation. The proposed “dual-domain comprehension” model further divides artistic comprehension into two domains: “professional comprehension” and “experiential comprehension.” The former embodies abstract reasoning and symbolic processing, while the latter focuses on emotional association and context-dependent processing. This model not only echoes Craik and Lockhart's (1972) deep processing theory but also extends Paivio's (1986) dual coding theory, revealing that figurative works are more easily accessible to narrative and language channels, while the comprehension of abstract works relies on the support of background knowledge and symbolic systems (Hayn-Leichsenring et al., 2020; Durkin et al., 2025). Furthermore, this study validates Jauss's (1982) “expectational horizon” and Gadamer's (1989) “effect history” theory from the perspective of reception aesthetics, providing empirical support for the triadic relationship between “knowledge structure, cultural experience, and meaning generation” in comprehension.

At a practical level, this study offers preliminary insights for art education and exhibition design. Differentiated instruction may be considered based on disciplinary background. Art students may benefit from strengthened “context-symbolization” training, while non-art students may be supported in transitioning from perceptual to more structured understanding through contextual introduction and narrative scaffolding.

### 6.3 Methodological reflections and future directions

While this study established a relatively robust chain of evidence through a combination of qualitative and quantitative methods, limitations remain. First, the sample size was limited, focusing on undergraduates from a single university, and the generalizability of the results remains to be verified. Second, the discriminatory power and inter-variable interactions of some constructs (such as “emotion” and “comprehension barriers”) in quantitative measurement require optimization. While the structural equation model meets statistical standards, the dynamic mechanisms of the complex “context → emotion → comprehension” pathway require further investigation. Although disciplinary background was used as a proxy for artistic expertise in this study, expertise is inherently a multidimensional construct. Future research could incorporate more fine-grained measures, such as years of training, frequency of art engagement, or visual literacy assessments, to better capture variations in expertise. It should be noted that perception was explored through qualitative prompts rather than standardized psychometric instruments, which may limit the precision of measurement. Additionally, there are certain limitations in the assessment of emotions in this study, as specific aesthetic emotions that may be evoked in artistic contexts were not included in the analysis. A further limitation concerns the applicability of the findings. As the present study did not include a fine-grained comparison between clearly defined expert and novice groups, the implications for developing tailored educational materials should be considered preliminary. Future research could explore three areas: First, experimental manipulation and multimodal tracking (such as eye movement, verbal, and psychophysiological indicators) could be used to examine the causal and temporal effects of the “context → diversion” mechanism. Second, dynamic Bayesian or hidden Markov models could be used to simulate the comprehension pathway and verify its generalizability in cross-cultural samples. Third, the “Dual Domain Comprehension Scale” could be optimized to construct a second-order factor structure and to test the effectiveness of interventions in educational and exhibition settings through RCTs. Overall, this study provides a new explanatory framework for the psychological mechanisms of art comprehension and lays a methodological foundation for intervention research in education and cultural communication.

## 7 Conclusion

This study's sample consisted of undergraduate students from a single institution, with a relatively concentrated age and educational background, limiting the generalizability of the conclusions. Audiences of different ages, occupations, and cultural backgrounds may exhibit differences, requiring further verification. Furthermore, this study's cross-sectional self-reporting approach makes it difficult to reveal the dynamics and causal mechanisms of understanding. Future work could experimentally manipulate the type and timing of background information, incorporating tracking techniques such as eye movements and real-time interviews, to examine the causal effects of the “context-to-understanding diversion.” Furthermore, expanding the sample and media coverage, and refining the “professional understanding-experience understanding” indicator system, could enhance model fit.

In summary, this study systematically compared the different approaches to understanding abstract and figurative art across different groups, confirming the pivotal and diverging role of contextual factors and revealing two stable styles of understanding: professionalization and experientialism. Qualitative and quantitative findings corroborate each other, and practical strategies for teaching and exhibition are proposed, providing empirical support for enhancing audiences' understanding of art.

## Data Availability

The original contributions presented in the study are included in the article/Supplementary material, further inquiries can be directed to the corresponding author.
