# Supplementary material for: When the image loses its shape: a comparative study of college students' understanding pathways of abstract and figurative art
Source: Front Psychol. 2026 May 18;17:1762388. doi: 10.3389/fpsyg.2026.1762388 (PMC13225768; doi:10.3389/fpsyg.2026.1762388)
Supplement: Supplementary file 1 [file Supplementary_file_1.docx]

## **Appendix A: Qualitative Interview Outline**

This study's interview outline, designed based on the research objectives and theoretical framework, aimed to explore the understanding paths of senior undergraduates at a comprehensive university when viewing abstract and figurative artworks, and to compare the differences between students from art and non-art colleges. The interview questions were organized into five dimensions, each corresponding to a specific research objective or theoretical basis.

1. Perception Dimension

What was your first impression of this work?

In the work, what elements first caught your attention (such as color, shape, characters, etc.)?

2. Understanding Dimensions

What meaning or theme do you think this work conveys?

How did you come to this view step by step?

Compared with other types of works (abstract/figurative), what differences do you think there are in the difficulty of understanding?

3. Emotional Dimension

What emotions or psychological feelings do you have when viewing this work?

Do these emotions influence your understanding of the work? If so, how?

4. Background Dimension

Do you think your disciplinary background (art/non-art) influences the way you understand this type of work?

Did you have any artistic education or training? How has this experience influenced your understanding?

5. Comprehensive Reflection Dimension

In your opinion, what are the most important factors in understanding a work of art? (The work itself, personal experience, education, external interpretation, etc.)

Which do you think is more likely to make you think: abstract art or figurative art? Why?

## **Appendix B : Quantitative Research Phase Questionnaire**

| **Dimensions** | **Sub-dimension** | **Item number** | **Example item** |
| --- | --- | --- | --- |
| **Perception** | **visual perception** | A1 | The first thing I notice is the color combination in the picture. |
|  |  | A2 | The contrast between light and dark colors will attract my strong attention. |
|  |  | A3 | The color of the picture will affect my first impression of the work. |
|  |  | A4 | I notice the direction and repetition of lines in a work. |
|  |  | A 5 | There are shapes or outlines in a piece that grab my attention. |
|  | **Intuitive identification** | A 6 | I was able to quickly identify the style and subject matter of this figurative artwork. |
|  |  | A 7 | I can quickly understand what the work is trying to express. |
|  |  | A 8 | Through the shapes and colors of the work, I can intuitively understand the content of the work. |
|  |  | A9 | I can quickly identify the art genre to which this work belongs . |
| **emotional response** | **Positive emotions** | B1 | This work makes me feel happy or comfortable. |
|  |  | B2 | I appreciate the work for its beauty. |
|  |  | B3 | When I look at the works, I feel relaxed. |
|  | **negative emotions** | B4 | This work makes me feel depressed or uneasy. |
|  |  | B5 | I may feel anxious or nervous about the content of my work. |
|  |  | B6 | Certain works make me feel alienated or uncomfortable. |
|  | **Mixed emotions** | B7 | I have conflicting emotions when viewing the work. |
|  |  | B8 | The work delighted and confused me at the same time. |
|  |  | B9 | I feel like the work evokes a variety of emotions that are difficult to put into words. |
| **understand** | **Lifestyle Association** | B10 | This work reminds me of my personal life experiences. |
|  |  | B11 | I associate the work with familiar memories. |
|  |  | B12 | The work reminds me of certain real people or things. |
|  | **Artistic Identification** | C1 | I can identify the artistic style or genre to which the work belongs. |
|  |  | C2 | I can tell if a piece is abstract or figurative. |
|  |  | C3 | I can recognize the artistic techniques embodied in the work. |
|  | **Subjective speculation** | C4 | I try to construct a story through the content of my work. |
|  |  | C5 | I will imagine the events or character relationships behind the work. |
|  |  | C6 | I will connect the visual elements into a complete narrative. |
|  | **Abstract reasoning** | C7 | I will understand the deeper meaning of the work through logical reasoning. |
|  |  | C8 | I think about the abstract relationships between the elements in my work. |
|  |  | C9 | I would interpret the work as a metaphor or philosophical expression. |
|  | **Symbolic interpretation** | C10 | I can associate the elements of the picture with certain symbolic meanings. |
|  |  | C11 | I will try to explain the symbols or metaphors in the work. |
|  |  | C12 | I would understand the work as a symbol of some social or cultural significance. |
|  | **Comprehension barriers** | C13 | I couldn't understand this work and found it difficult to comprehend. |
|  |  | C14 | The subject of this work is unclear and I have difficulty understanding what it is expressing. |
|  |  | C15 | I was confused when I looked at this work and didn't know how to interpret it. |
|  | **Academic Narrative** | C16 | I will use my knowledge of art history to explain the meaning of this work. |
|  |  | C17 | I can identify the cultural and historical context of a work and interpret it based on that. |
|  |  | C18 | I use theoretical frameworks to support my understanding of artworks. |
| **Background factors** | **Discipline Background** | D1 | The professional knowledge I have learned helps me understand works of art . |
|  |  | D2 | My academic training makes it easier for me to understand art. |
|  |  | D3 | My experience in art education has enabled me to appreciate the work in depth. |
|  | **Personal experience** | D 4 | I relate my work to my daily life. |
|  |  | D 5 | My upbringing influences my understanding of the work. |
|  |  | D 6 | I will understand the work based on my own interests or hobbies. |
| **xPerception​** | **x Visual Perception** | x A1 | The first thing I notice is the color combination in the picture. |
|  |  | x A2 | The contrast between light and dark colors will attract my strong attention. |
|  |  | x A3 | The color of the picture will affect my first impression of the work. |
|  |  | x A4 | I notice the direction and repetition of lines in a work. |
|  |  | x A 5 | There are shapes or outlines in a piece that draw my attention. |
|  | **xIntuitive recognition** | x A 6 | I was able to quickly identify the style and subject matter of this figurative artwork. |
|  |  | x A 7 | I can quickly understand what the work is trying to express. |
|  |  | x A 8 | Through the shapes and colors of the works, I can intuitively understand the content of the works. |
|  |  | xA9 | I can quickly identify the art genre to which this work belongs . |
| **xEmotional response** | **x Positive emotions** | x B1 | This work makes me feel happy or comfortable. |
|  |  | x B2 | I appreciate the work for its beauty. |
|  |  | x B3 | When I look at the works, I feel relaxed. |
|  | **xNegative emotions** | x B4 | This work makes me feel depressed or uneasy. |
|  |  | x B5 | I may feel anxious or nervous about the content of my work. |
|  |  | x B6 | Certain works make me feel alienated or uncomfortable. |
|  | **x Complex emotions** | x B7 | I have conflicting emotions when viewing the work. |
|  |  | x B8 | The work delighted and confused me at the same time. |
|  |  | x B9 | I feel like the work evokes a variety of emotions that are difficult to put into words. |
| **xUnderstanding​** | **x Lifestyle Association** | x B10 | This work reminds me of my personal life experiences. |
|  |  | x B11 | I associate the work with familiar memories. |
|  |  | x B12 | The work reminds me of certain real people or things. |
|  | **x Art Recognition** | x C1 | I can identify the artistic style or genre to which the work belongs. |
|  |  | x C2 | I can tell if a piece is abstract or figurative. |
|  |  | x C3 | I can recognize the artistic techniques embodied in the work. |
|  | **x Narrative Construction** | x C4 | I try to construct a story through the content of my work. |
|  |  | x C5 | I will imagine the events or character relationships behind the work. |
|  |  | x C6 | I will connect the visual elements into a complete narrative. |
|  | **xAbstract reasoning** | x C7 | I will understand the deeper meaning of the work through logical reasoning. |
|  |  | x C8 | I think about the abstract relationships between the elements in my work. |
|  |  | x C9 | I would interpret the work as a metaphor or philosophical expression. |
|  | **Symbolic interpretation of x** | x C10 | I can associate the elements of the picture with certain symbolic meanings. |
|  |  | x C11 | I will try to explain the symbols or metaphors in the work. |
|  |  | x C12 | I would understand the work as a symbol of some social or cultural significance. |
|  | **xComprehension disorder** | xC13 | I couldn't understand this work and found it difficult to comprehend. |
|  |  | xC14 | The subject of this work is unclear and I have difficulty understanding what it is expressing. |
|  |  | xC15 | I was confused when looking at this work and didn't know how to interpret it. |
|  | **xAcademic Narrative** | xC16 | I will use my knowledge of art history to explain the meaning of this work. |
|  |  | xC17 | I can identify the cultural and historical context of a work and interpret it based on that. |
|  |  | xC18 | I use theoretical frameworks to support my understanding of artworks. |
| **x Background factors** | **x Subject Background** | x D1 | The professional knowledge I have learned helps me understand works of art . |
|  |  | x D2 | My academic training makes it easier for me to understand art. |
|  |  | x D3 | My experience in art education has enabled me to appreciate the work in depth. |
|  | **xPersonal experience** | x D 4 | I relate my work to my daily life. |
|  |  | x D 5 | My upbringing influences my understanding of the work. |
|  |  | x D 6 | I will understand the work based on my own interests or hobbies. |

## **Appendix C: Open Coding**

| **Respondent** | **Original expression** | **Preliminary coding** | **Dimension classification** |
| --- | --- | --- | --- |
| A3 | “The colors of this painting remind me of a repressed mood.” | Color and emotion | Perception/Emotion |
| A6 | "Judging from the repetition of lines, the author may have intended to convey a sense of order." | Formal reasoning | understand |
| N4 | "I have no idea what it means. It seems to be just some doodles." | Comprehension barriers | understand |
| N8 | "I think it looks like the scenery of my hometown, so it feels very familiar." | Lifestyle Association | Emotion/Background |
| A9 | “My background in art history made me realize that this was expressionism.” | Academic Narrative | background |
| A2 | “It immediately reminded me of Impressionist composition.” | Artistic Identification | understand |
| N6 | “This painting reminds me of playing in the fields as a child.” | Emotional association | emotion |
| A7 | "The contrast of colors seems to suggest the opposition between life and death." | Symbolic interpretation | understand |
| N2 | “These blocks of color look bright, but I’m not sure what they represent.” | Perception confusion | Perception/Understanding |
| A11 | "I learned similar techniques in art class before, so it's very familiar to me." | Educational experience adjustment | background |
| N5 | "I guess it's freedom of expression, but there's no particular basis for it." | Subjective speculation | understand |
| A4 | “The details are very delicate, and I think the author put a lot of effort into it.” | Composition/Detail Observation | Perception |
| N7 | “It looked like a forest, so I understood the content pretty quickly.” | Intuitive identification | understand |
| A10 | “This strong brushstroke gives me a sense of tension.” | Brushstrokes and emotions | Perception/Emotion |
| A12 | "The colors used in this painting are very dark, giving it a heavy feeling." | Color and Mood | Perception/Emotion |
| N1 | “I felt a sense of oppression from the chaos of the work.” | Form feeling | Perception/Emotion |
| A5 | “This painting has a strong sense of space, as if I could step into it.” | Spatial Perception | Perception |
| N3 | “I feel overwhelmed by these graphics.” | Form confusion | Perception/Understanding |
| A13 | “This abstract approach makes me feel uneasy, but also very attractive.” | Abstract Expressionism | emotion |
| N9 | "This is the strangest painting I've ever seen. I can't understand its purpose." | Difficult to understand | understand |
| A14 | “The author uses a variety of media to present a rich tapestry of emotions.” | Multimedia performance | Perception/Emotion |
| N10 | “I can understand the painting, but I’m not sure it conveys the emotion the author intended.” | Understanding Reflection | understand |
| A15 | "From the transition of colors, the painting seems to tell a sad story." | Color and Narrative | Understanding/Emotion |
| A16 | “The dynamic lines of this work give me a sense of movement.” | Dynamic Perception | Perception |
| N11 | “The work looks very abstract and it’s hard for me to interpret it.” | Abstraction is difficult to understand | understand |
| A17 | “The complexity of the painting confused me at first.” | Complexity response | Perception |
| N12 | "It seems like this painting is about expressing inner emotions, not necessarily having a clear graphic image." | Emotional expression | understand |
| A18 | “I can see the artist’s emotional expression from the way the work is expressed.” | Expression and emotion | Perception/Emotion |
| N13 | “The irregular shapes in the painting remind me of chaotic emotions.” | Form and emotional association | Perception/Emotion |
| A19 | “The work’s strong contrasting colors evoke a strong emotional response in me.” | Color contrast and emotion | Perception/Emotion |
| N14 | “Without an art background, it’s hard for me to understand these abstract expressions.” | Lack of background knowledge | background |
| A20 | “I can feel the passion and intensity in this work.” | Passionate performance | emotion |
| N15 | "I thought the painting made me feel very sad, but I didn't understand its deeper meaning." | Differences in feeling and understanding | Understanding/Emotion |
| A21 | “The arrangement of these lines reminds me of some kind of orderly structure.” | Perception of formal order | Perception |
| N16 | “The combination of color and light and shadow makes me feel that the work is full of unease.” | Color and light | Perception/Emotion |
| A22 | "The painting gives the impression of loneliness and isolation." | Loneliness | emotion |
| N17 | “The work has strong contrasts and displays a strong conflict.” | Contrast and conflict | Perception/Emotion |
| A23 | “This work reminds me of some situations I’ve experienced.” | Life experience association | Background/Emotion |
| N18 | “The distribution of these shapes gives me a sense of imbalance.” | Imbalance in form | Perception |
| A24 | “The colors used in this painting are very powerful and give me a strong sense of impact.” | Color Impact | Perception/Emotion |
| N19 | “I think this painting is very layered and each element is meaningful.” | Levels and meaning | understand |
| A25 | “The asymmetry in the painting disturbed me.” | Asymmetric form | Perception/Emotion |
| N20 | “There were parts of the work that left me with a strong sense of uncertainty.” | uncertainty | emotion |
| A26 | “The combination of color blocks makes the whole picture look very vibrant.” | Color block splicing and vitality | Perception/Emotion |
| N21 | "I was a little overwhelmed by this painting; it felt like every element was breaking the mold." | Breakthrough in form | Perception/Understanding |
| A27 | “The curves of the work are smooth and elegant, as if telling a heartwarming story.” | Curves and Narratives | Perception/Emotion |
| N22 | “The picture is too complicated and has too many details, so I can’t grasp the main point.” | Detail overload | Perception/Understanding |
| A28 | "The author creates a strong visual impact through contrasting colors." | Color contrast | Perception |
| N23 | "From the structure of the image, I feel that this painting expresses a kind of inner turmoil." | Structure and emotion | Understanding/Emotion |
| A29 | “The tones of the work suggest sadness and silence.” | Tone and Mood | Perception/Emotion |
| N24 | “The shapes and lines of this painting reminded me of my life experiences.” | Life Association | Background/Emotion |
| A30 | "I feel like these colors represent the contrast between life and death." | Color symbolism | Understanding/Emotion |
| N25 | “This painting gave me a strong visual experience, but it was difficult to understand the meaning behind it.” | The gap between visual perception and understanding | Perception/Understanding |

## **Appendix D : Descriptive Statistics Basic Indicators**

Descriptive statistical basic indicators

| name | Sample size | Minimum | Maximum | Average value | Standard Deviation | Median​ |
| --- | --- | --- | --- | --- | --- | --- |
| Artistic Identification | 665 | 1.000 | 5.000 | 3.384 | 0.820 | 3.333 |
| Background factors | 665 | 1.000 | 5.000 | 3.473 | 0.754 | 3.500 |
| Academic Narrative | 665 | 1.000 | 5.000 | 3.155 | 0.866 | 3.000 |
| Comprehension barriers | 665 | 1.000 | 5.000 | 3.317 | 0.828 | 3.333 |
| Symbolic interpretation | 665 | 1.000 | 5.000 | 3.473 | 0.813 | 3.667 |
| Abstract reasoning | 665 | 1.000 | 5.000 | 3.387 | 0.858 | 3.333 |
| Narrative Construction | 665 | 1.000 | 5.000 | 3.316 | 0.877 | 3.333 |
| Lifestyle Association | 665 | 1.000 | 5.000 | 3.151 | 0.917 | 3.000 |
| Emotionally driven | 665 | 1.000 | 5.000 | 3.229 | 0.676 | 3.111 |
| Intuitive identification | 665 | 1.000 | 5.000 | 3.261 | 0.840 | 3.250 |
| visual perception | 665 | 1.000 | 5.000 | 3.766 | 0.651 | 3.800 |
| xSubjective speculation | 665 | 1.000 | 5.000 | 3.416 | 0.849 | 3.333 |
| x Art Recognition | 665 | 1.000 | 5.000 | 3.447 | 0.833 | 3.667 |
| x Lifestyle Association | 665 | 1.000 | 5.000 | 3.290 | 0.867 | 3.333 |
| x Background factors | 665 | 1.000 | 5.000 | 3.523 | 0.769 | 3.500 |
| xAcademic Narrative | 665 | 1.000 | 5.000 | 3.292 | 0.864 | 3.000 |
| xComprehension disorder | 665 | 1.000 | 5.000 | 3.056 | 0.900 | 3.000 |
| Symbolic interpretation of x | 665 | 1.000 | 5.000 | 3.458 | 0.825 | 3.333 |
| xEmotional Drive | 665 | 1.000 | 5.000 | 3.168 | 0.720 | 3.111 |
| xIntuitive recognition | 665 | 1.000 | 5.000 | 3.583 | 0.806 | 3.750 |
| x Visual Perception | 665 | 1.000 | 5.000 | 3.779 | 0.704 | 4.000 |
| xAbstract reasoning | 665 | 1.000 | 5.000 | 3.387 | 0.821 | 3.333 |
